# Transmission models indicate Ebola virus persistence in non-human primate populations is unlikely

**DOI:** 10.1098/rsif.2021.0638

**Published:** 2022-02-02

**Authors:** David T. S. Hayman, Reju Sam John, Pejman Rohani

**Affiliations:** ^1^ Molecular Epidemiology and Public Health Laboratory, Hopkirk Research Institute, School of Veterinary Science, Massey University, Private Bag 11 222, Palmerston North 4442, New Zealand; ^2^ Te Pūnaha Matatini, Centre for Research Excellence, Auckland, New Zealand; ^3^ Odum School of Ecology, University of Georgia, Athens, GA 30602, USA; ^4^ Center for Influenza Disease & Emergence Research, University of Georgia, Athens, GA 30602, USA; ^5^ Department of Infectious Diseases, College of Veterinary Medicine, University of Georgia, Athens, GA 30602, USA

**Keywords:** Ebola virus disease, infection dynamics, reservoir hosts, epidemiological modelling

## Abstract

Infectious diseases that kill their hosts may persist locally only if transmission is appropriately balanced by susceptible recruitment. Great apes die of Ebola virus disease (EVD) and have transmitted ebolaviruses to people. However, understanding the role that apes and other non-human primates play in maintaining ebolaviruses in Nature is hampered by a lack of data. Recent serological findings suggest that few non-human primates have antibodies to EVD-causing viruses throughout tropical Africa, suggesting low transmission rates and/or high EVD mortality (Ayouba A *et al.* 2019 *J. Infect. Dis.* **220**, 1599–1608 (doi:10.1093/infdis/jiz006); Mombo IM *et al.* 2020 *Viruses* **12**, 1347 (doi:10.3390/v12121347)). Here, stochastic transmission models of EVD in non-human primates assuming high case-fatality probabilities and experimentally observed or field-observed parameters did not allow viral persistence, suggesting that non-human primate populations are highly unlikely to sustain EVD-causing infection for prolonged periods. Repeated introductions led to declining population sizes, similar to field observations of apes, but not viral persistence.

## 1. Background

Ebola virus disease (EVD) kills apes: both people and the great apes of Africa. Almost half of the EVD-infected people die and mortality from EVD is thought to have caused massive declines in Central African apes [1,2]. Infections that cause high mortality rates are thought to reduce their likelihood of onward transmission because death removes infected hosts [3,4]. This higher mortality, or pathogenicity, is one of the reasons so-called ‘spillover’ events of infectious agents from one species or group of species that form a reservoir to new hosts fail to persist in those new host populations. Global case-fatality studies for infectious diseases of domestic mammals suggest that the evolutionary distance from an infected host to other hosts is a strong predictor of disease-induced mortality [5]. Adaptation to one host may be detrimental for inter-host transmission [6] and it has been shown that more pathogenic emerging viral infections are on average less likely to establish transmission cycles within humans [7]. Infections are expected to optimize the level of virulence (which may include pathogenic effects) to optimize their fitness, often characterized through the basic reproductive number (*R*_0_) [4,8].

Virulence, however, may be adaptive and a number of mechanisms have been proposed to explain its evolution. For instance, in malaria-causing *Plasmodium* parasites, it has been shown that higher virulence is associated with increased within-host competitiveness [9]. Furthermore, while there may be limited disease in ancestral hosts, transmission and adaptation to a new host does not necessarily lead to avirulence or low pathogenicity, as exemplified by the adaptation of Simian immunodeficiency viruses (SIVs; with low mortality in non-human primates [10]) to people (human immunodeficiency virus (HIV); with high mortality in people but with continued transmission). Indeed, infections such as rabies viruses may persist in populations despite 100% mortality [11]. In the case of rabies and HIV, however, sometimes prolonged incubation periods and persistent infection prior to death, respectively, reduce the likelihood of pathogen fade-out in populations. Recent genomic evidence suggests that there has been Ebola virus latency and recrudescence of Ebola virus infection in humans, posing the question of what that might mean for persistence in wild species [12,13].

Four different viruses from four *Ebolavirus* species have caused EVD in Africa: Ebola virus (EBOV), Sudan virus (SUDV), Bundibugyo virus (BDBV) and Tai Forest virus (TAFV), with all but TAFV having caused fatal human disease. Outbreaks in people are sporadic, though the frequency and the size of outbreaks in particular may be increasing. The largest EVD outbreak in West Africa from 2013 to 2016 killed 11 310 of 28 616 cases [14]; the second-largest outbreak was in the Democratic Republic of Congo (DRC) from 2018 to 2020, with 3470 EVD confirmed (3317) and probable cases, with 2287 deaths recorded (overall case-fatality ratio 66%) [15,16].

**Figure 1.**
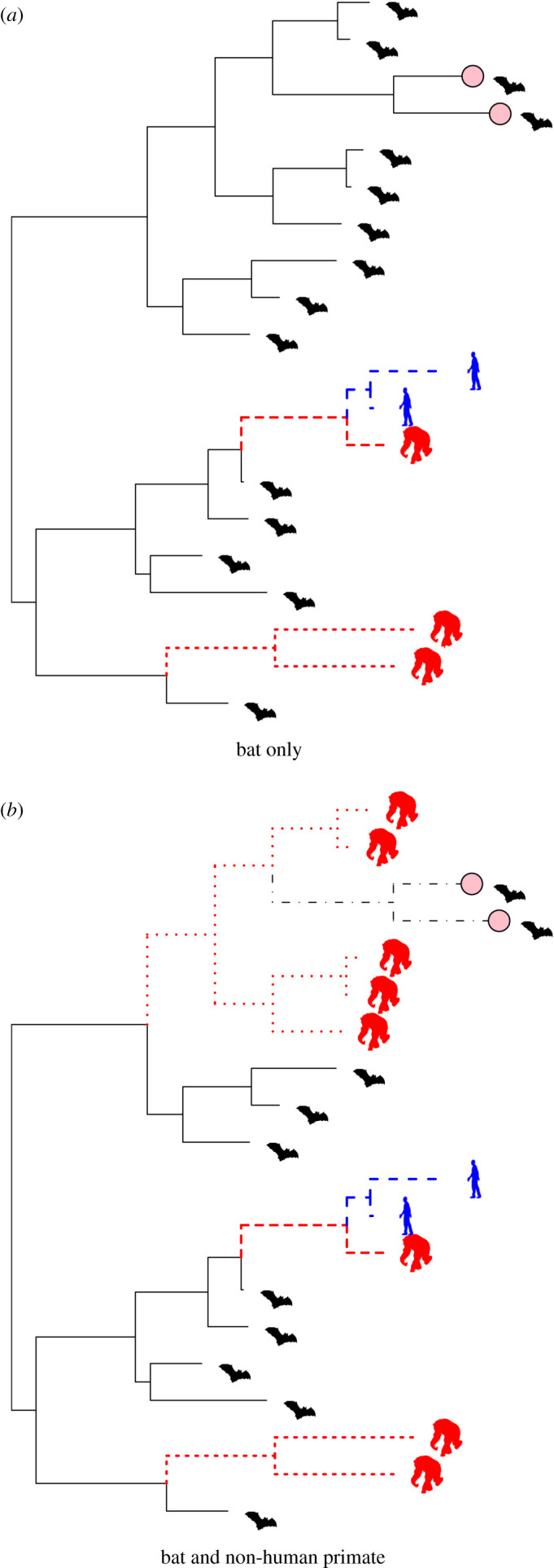
Potential EVD transmission between mammalian Orders. Extant sylvatic viruses (here in bats) are pink tip-filled circles. In (*a*), bats are the only reservoir hosts allowing long-term viral persistence and infection switching into primates (including humans) is irreversible and fails to persist, whereas in (*b*) viruses reversibly switch between mammalian Orders.

Ebola virus RNA has been isolated from dead apes and anti-EBOV antibodies have been detected in apes and other non-human primates [17–20]. The presence of antibodies suggests that non-lethal infections can occur, but the prevalence of antibodies is low, suggesting that this is rare [17,18,20]. Experimental studies confirm non-human primate susceptibility to fatal EBOV infection [21]. Apes, monkeys and antelopes have died during outbreaks in wildlife, with substantial population declines observed in their populations [22]. By contrast, ecological and experimental studies suggest that EBOV infection in bats may be non-fatal, with a likely short-lived infection that induces antibodies [23–26]. Together, these data have led to hypotheses that bats are the maintenance reservoir host for EVD-causing viruses, while non-human primates are the victims of EVD spillover events, also acting as intermediate hosts for onward transmission to people [17,27] (figure 1).

Phylogenetic models of EBOV place the first viruses isolated in 1976 from DRC near the root of the EBOV phylogenetic tree, suggesting that all other known outbreaks descended from a closely related virus [28]. Key issues for understanding sylvatic maintenance in wildlife are that the number of viral sequences is limited, preventing analyses determining if viruses discovered in primates are dead-ends or if they contribute to the evolution of successive generations of ebolaviruses (figure 1) [29].

Compartmental models used for the mathematical modelling of infectious diseases are highly flexible tools, allowing changes to model structures (such as including incubation periods) and parameters (such as infection-induced mortality) to test different hypotheses. In particular, they allow investigation into the impacts of variation in model structure and parameterization on outbreak probability (e.g. if the basic reproduction number, *R*_0_, i.e. the expected number of cases from one infected case in a population where all individuals are susceptible to infection, is >1), persistence (e.g. endemic) and the related infection dynamics (e.g. seasonality) [30]. Stochastic models are particularly useful for understanding stochastic processes, like disease transmission and extinction. Moreover, when $R_{0}⪆1$, as for Ebola virus in people [31–33], stochastic models predict bimodal final epidemic sizes that probably better capture the probabilistic nature of transmission than deterministic models [34,35].

Here, we formulate probabilistic transmission models [30,36] of EVD in a simulated non-human primate population to examine whether stochastic viral fade-out or long-term persistence occur within a range of reasonable case-fatality rates, and to establish which mechanisms are consistent with evidence from the field. These models can help explain field observations if they successfully recapitulate empirical findings and help inform future monitoring, should they predict long-term viral persistence [37], which is useful in low-resource and difficult field settings. The models comprised different assumptions regarding the incubation period, the timing of disease-induced mortality, host demography (faster versus slower turnover) and implementation of viral importation. We have illustrated our results with a baseline model using parameters that are likely to represent African great apes (gorillas and chimpanzees), highlighting key differences when needed.

## 2. Material and methods

Here, we formulate a basic Susceptible–Infected–Recovered (*SIR*) model for non-human primates, based on field observations and experimental infection data for non-human primates [21] and, when missing key parameters, from clinical reports for humans [38]. The basic *SIR* model structure incorporates demography (*μ*), disease-induced mortality (*ρ*) and external force of infection (*ε*) and infectious imports from external sources (*δ*).

We classified the entire population into three compartments, namely *SIR*. Let *S*, *I* and *R* represent the number of susceptible, infected and recovered classes of non-human primates at time *t*. Also assume that the total number of the population is *N* = *S* + *I* + *R*. Furthermore, we assume a homogeneous mixing of individual primates within the population. The rate of change of susceptible hosts *S*(*t*) at time *t* will be

2.1 $$\begin{array}{rl} \frac{dS}{dt} & =\text{new members into the }S\text{ compartment (i.e. birth)}\\ & \;-\text{loss due to transmission and natural mortality}. \end{array}$$

New members added to the *S* compartment will be equal to the product of the per capita birth rate (*μ*) and the total number of population (*N*), i.e. *μN*. There are three ways a susceptible individual can leave the susceptible compartment:

(i) Interaction between the susceptible individuals and the infected individuals. Since the proportion of infectious contacts is *I*/*N*, disease transmission will be proportional to $\frac{I}{N}\times S$. If *β* is the transmission rate, the rate at which susceptible individuals leave the susceptible compartment and enter the infected compartment will be $\beta \frac{SI}{N}$.
(ii) Susceptible individuals become infected from an external source. If the interaction rate is *ε*, susceptibles enter the infectious class at rate *εS*.
(iii) Susceptible individuals can die from natural causes. If the death rate is *μ*, a rate *μS* of susceptibles leave the susceptible compartment per day.

These considerations may lead to the following equation:

2.2 $$\frac{dS}{dt}=\mu N-\beta \frac{SI}{N}-\epsilon S-\mu S.$$

In the above equation, we assume frequency-dependent transmission, i.e. the number of contacts is independent of population size [30]. The disease-transmitting contacts will be determined by social factors. If population size (or, precisely, the density of individuals) determines the contact rate then the disease transmission rate will be proportional to *I* × *S*. If *β* is the transmission rate, the rate at which susceptible individuals leave the susceptible compartment and enter the infected compartment will be *βSI*. Such interactions are known as density-dependent interactions.

To encapsulate frequency- and density-dependent transmission into a single equation, we introduce a dimensionless scaling index, *q*. The generalized equations for the rate of change of susceptible hosts *S*(*t*), infected hosts *I*(*t*) and recovered hosts *R*(*t*) at time *t* are

2.3 $$\begin{array}{rl} \frac{dS}{dt} & =\mu N-\beta \frac{SI}{N^{q}}-\epsilon S-\mu S,\\ \frac{dI}{dt} & =\underset{\mathrm{contribution}\;\mathrm{towards}\;I}{\underbrace{\frac{\beta SI}{N^{q}}+\epsilon S+\delta }}-\overset{\mathrm{disease-induced death}}{\overbrace{\rho \gamma I}}-\underset{\mathrm{recovery}\;\mathrm{rate}}{\underbrace{\gamma (1-\rho )I}}-\overset{\mathrm{natural}\;\mathrm{death}}{\overbrace{\mu I}},\\ \frac{dR}{dt} & =\gamma (1-\rho )I-\mu R, \end{array}\}$$

where

2.4 $$q=\left\{ \begin{array}{cc} 1, & \text{frequency dependent}\\ 0, & \text{density dependent}\\ <1\;\text{and}\;>0, & \text{in between frequency and density dependent}. \end{array}\right.$$

The *δ* in equation (2.3) represents the influx of infectious individuals from another host population, which is independent of *S*(*t*) and *I*(*t*). All variables and parameters are summarized in table 1.

**Table 1.** Parameters and variables

	description	values	units	references
*N*	total population	*S* + *I* + *R*	n.a.	[39,40]
*S*	number of susceptible at time *t*	n.a.	n.a.	n.a.
*I*	number of infected at time *t*	n.a.	n.a.	n.a.
*R*	number of recovered at time *t*	n.a.	n.a.	n.a.
*S*(0)	initial susceptible	10 000	n.a.	n.a.
*I*(0)	initial infected	1	n.a.	n.a.
*R*(0)	initial recovered	0	n.a.	n.a.
*μ*	birth and death rate	5 × 10^−3^–10^−4^	per capita day^−1^	[41]
*β*	transmission coefficient	0.1–2	per capita day^−1^	[21,42–45]
*γ*	recovery rate	0.1	day^−1^	[21,42–45]
*ρ*	disease-induced case fatality	0–0.9	day^−1^	[1,22,41,45–47]
*ε*	external force of infection rate	2 × 10^−5^	day^−1^	[22,48]
*δ*	new external infection rate	0.01	day^−1^	[22,48]

Ranges of values are given when used. The SIR model structure is shown in equations (2.3) and (2.5). The total population size remained the same for *SEIR* (*E* = Exposed) models, but was *S* + *E* + *I* + *R*, whereas it was double in coupled meta-population models. Birth and death rates of 5 × 10^−4^ were used unless stated. See the electronic supplementary material for further parameters.

Demographic stochasticity was incorporated through the implementation of Gillespie’s *τ*-leap algorithm [49], using adapted R functions [50,51]. Here, a time step *τ* is chosen and at each step the number of times an event occurs is given by a Poisson distribution with the mean determined by equation (2.5),

2.5 $$\begin{array}{rl} & P_{\mathrm{birth}}=\text{Poisson}(\tau \times \mu N),\\ & P_{\mathrm{infection}}=\text{Poisson}(\tau \times \beta SI/N{\;}^{q}),\\ & P_{\mathrm{recovery}}=\text{Poisson}(\tau \times \gamma \times (1-\rho )I),\\ & P_{\mathrm{death}\;\mathrm{disease}}=\text{Poisson}(\tau \times \rho \times \gamma I),\\ & P_{{\mathrm{death}}_{S}}=\text{Poisson}(\tau \times \mu S),\\ & P_{{\mathrm{death}}_{I}}=\text{Poisson}(\tau \times \mu I),\\ & P_{{\mathrm{death}}_{R}}=\text{Poisson}(\tau \times \mu R),\\ & P_{{\mathrm{immigration}}_{I}}=\text{Poisson}(\tau \times \delta )\\ \;\mathrm{and}\; & P_{\mathrm{external}\;\mathrm{infection}}=\text{Poisson}(\tau \times \epsilon S). \end{array}\}$$

Simulations were run 100 times for each parameter combination for 20 years, with the initial introduction of one infectious individual into a totally susceptible population at time zero.

Specific assumptions were then tested. First, while all primates are relatively long-lived and have similar birthing and mortality patterns, monkey and other non-human primate life cycles are faster than apes [52], so we tested if virus population persistence was increased with a 10-fold increase in demographic turnover (table 1). Alternative model structures were then developed where: (i) an incubation period *ϕ* was introduced to model an exposed (*E*) class with an average 9-day incubation period to form an *SEIR* model (see electronic supplementary material, equation (7)); (ii) mortality (*ρ*) was moved from the infected *I* class to the immune *R* class to represent late mortality and limit the impact of mortality on transmission and *R*_0_ (see the electronic supplementary material), or which also captures potential transmission from corpses, the contact with which has been a risk factor for human infection [22,53,54], by maintaining the infectious period duration, but removing the individual; (iii) re-introduction of the virus was simulated via either a fixed force of infection ($S_{\epsilon }$) or an influx of an infectious individual (e.g. from another putative host or delayed sexual transmission (*δ*, see the electronic supplementary material); (iv) a second population of 10 000 with migration between the populations was simulated with an *SIR* structure and mortality in the *I* class, and, finally; (v) spatial and meta-population structure. Previously, spatial structure and its impact on infection dynamics in non-human primates has been modelled using a biased random walk step on a 51 × 51 lattice with small family groups (10) with internal *SIR* dynamics [55]. Here, we introduced spatial and meta-population structure following [56], with 1600 sub-populations (nodes) of approximately 200 individuals, with each node having either an *SIR* or *SEIR* structure, with no disease-induced mortality, which is the most likely scenario to allow persistence. Varying spatial structure was implemented through increasing coupling from very low (0.1) to very high (0.9) among proximal nodes on a meta-population network [56] (see the electronic supplementary material). All models and reproducible R code are available at: https://github.com/dtsh2/ebola_model.

## 3. Results

**Figure 2.**
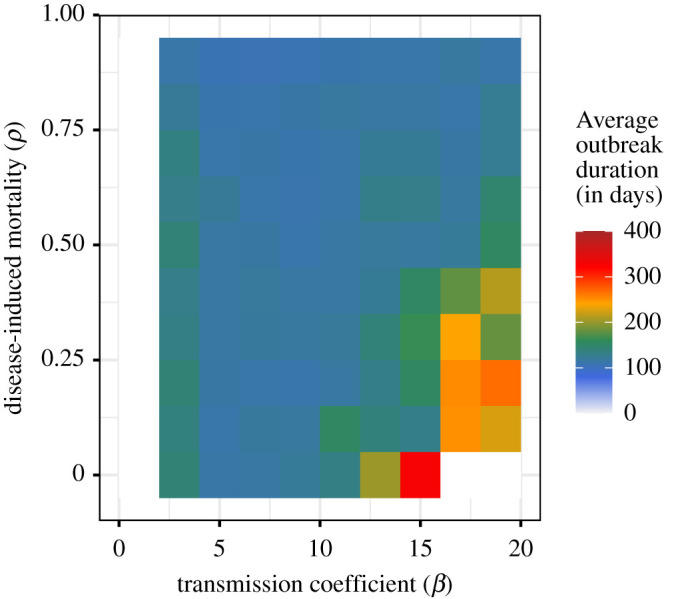
Ebola virus disease outbreak duration.

Simulations of the baseline *SIR* model (equation (2.3)) for EVD outbreaks in apes with single introductions of virus for a range of transmission rates (hypothetical *R*_0_, ranging from 1 to 20) and the proportion of cases that die (infection fatality rate, *ρ*, from 0% to 90% fatality) indicate that outbreaks are only sustained for more than half a year in populations with high transmission rates and very low case-fatality rates (figure 2). Increased case-fatality rates effectively reduce *R*_0_, because it reduces the infectious period in this model (see the electronic supplementary material).

**Figure 3.**
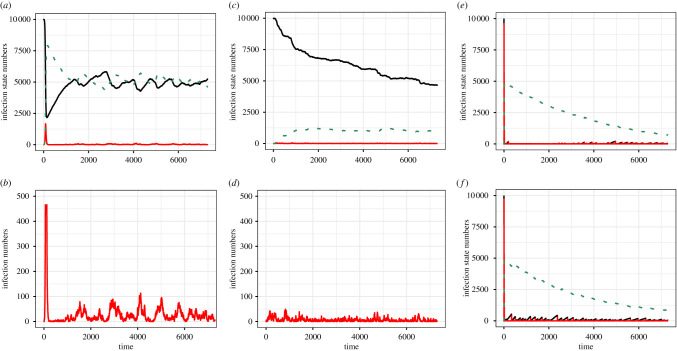
Ebola virus disease dynamics. Simulations were of an SIR model over 20 years in long-lived non-human primate populations, with repeated introductions of viruses. Panel (*a*,*c*) show susceptible (black), infected (red) and immune (green dashes) numbers. Panel (*b*,*d*) show infected numbers only for the same simulations as above on a limited scale (max. 500). Panel (*e*,*f*) show susceptible (black), infected (red) and immune (green dashes) numbers of the model with *q* = 0.0, density dependent and *q* = 0.5, mix of density and frequency dependent, respectively. Disease-induced mortality (*ρ*) was set to be 0.5. Time is in days. (*a*) *S* (black), *I* (red), *R* (green) versus time (in days) with no disease-induced mortality and *q* = 1.0. (*b*) *I* (red) versus time (in days) with no disease-induced mortality and *q* = 1.0. (*c*) *S* (black), *I* (red), *R* (green) versus time (in days) with disease-induced mortality and *q* = 1.0. (*d*) *I* (red) versus time (in days) with disease-induced mortality and *q* = 1.0. (*e*) *S* (black), *I* (red), *R* (green) versus time (in days) with disease-induced mortality (*ρ*) and *q* = 0.0. (*f*) *S* (black), *I* (red), *R* (green) versus time (in days) with *ρ* = 0.5 and *q* = 0.5.

**Figure 4.**
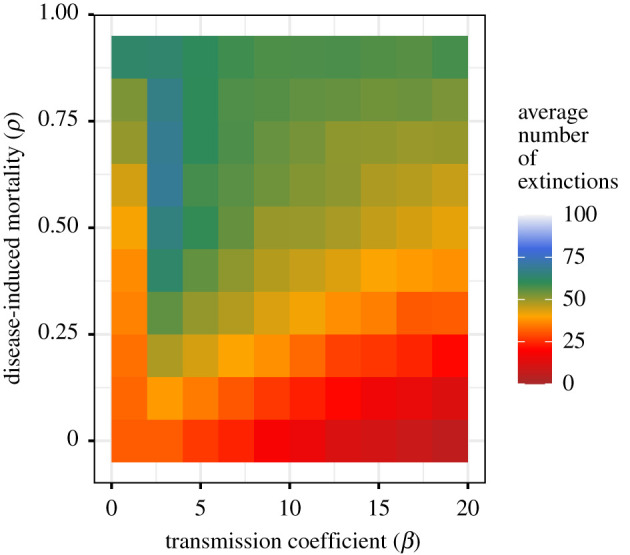
Ebola virus disease extinction frequency with re-introduction of virus. Disease-induced mortality (*ρ*) ranges from 0 to 0.9 and the transmission coefficient (*β*) from 1 to 20. Each cell is 100 simulations of our model: equation (2.3). Duration is in days. In figure 4, infection was introduced stochastically at least approximately three times annually over 20 years.

Given the failure of EVD to persist following single introductions, re-introduction of virus was simulated. Simulations were run for 20 years with varying case-fatality rates and repeated re-introductions of virus, either through infected individuals or through an external force of infection (e.g. from another putative host or delayed sexual transmission [13,57]) (equations (2.3) and (2.5), figure 3) [30]. All likely parameter values lead to pathogen fade-out, despite frequent re-introduction (figure 4). For example, there was still no long-term persistence following re-introduction using *R*_0_ ≈ 2, as estimated from human data [31,42,43,58], despite approximately three introduction events per year (figure 3).

However, repeated re-introductions resulted in two different patterns. With no case fatality, recovered immune individuals survived. Because of the longevity of the ape, these animals are present in the populations for long periods (figure 3*a*). With high case-fatality rates (e.g. 50%), however, few immune individuals survive and mortality reduces onward transmission (figure 3*c*). The initial and subsequent outbreaks in the population with low case fatality were larger. Furthermore, the population sizes remain similar, but declines are seen when disease-induced mortality is included (figure 3*c*,*d*). The serological patterns of very low seroprevalence in the high case-fatality rate model more closely match those seen in field data [17]. Here, we assumed frequency dependence (*q*=1 in equation (2.3)) for the number of contacts of infected non-human primates with other primates. We have further investigated the density dependence of the interactions by changing the value of *q* in equation (2.3). Figure 3*e* shows the disease dynamics with disease-induced mortality when density-dependence interaction between the non-human primates occurs (when *q*=0). Figure 3*f* refers to disease dynamics with disease-induced mortality (analogous to figure 3*c*,*e*) but the interaction between the non-human primates is assumed to be a mixture of density dependence and frequency dependence, i.e. *q*= 0.5. The model results with these transmission mechanisms (figure 3*e*,*f*) do not match with the field data [17,18] owing to the presence of a large number of apes with antibodies. This was true for a range of assumptions and so hereon we will be interested in models with a value of 1 assigned to *q* (frequency dependence).

**Figure 5.**
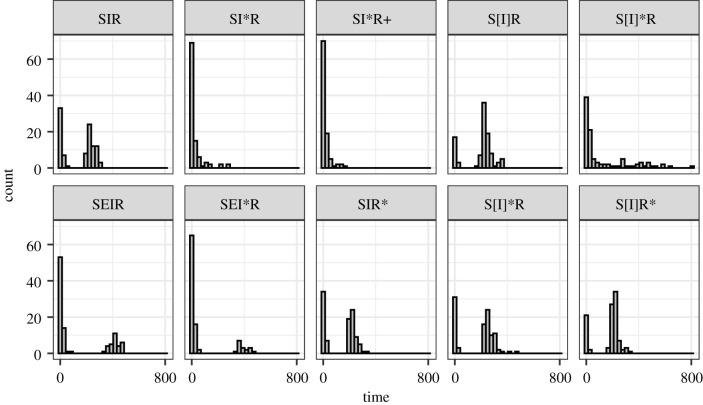
Frequency of extinction times for different model structures and assumptions. Model structures are susceptible (*S*), exposed (incubating, *E*), infectious (*I*) and recovered (immune, *R*). Square brackets [] represent model compartments to which infectious individuals were re-introduced, * when 50% disease mortality was incorporated, with the * position showing to which category it was applied (*I* versus *R*) and + representing a model with a 10-fold increase in the demographic rate.

We performed extensive sensitivity analyses. The results of pathogen fade-out were consistent across a range of model structures and assumptions, including 10-fold faster demographic rates or alternative assumptions (figure 5 and see the electronic supplementary material). Neither the introduction of another population with migration of infected individuals between them to make a meta-population nor the incorporation of an exposed category to allow for an incubation period (an *SEIR* network) altered the likelihood of EVD persisting in these model populations (figure 5 and see the electronic supplementary material). Large population declines occurred only when mortality was moved from the infected class (*I*) to the immune (recovered, *R*) class. This allowed mortality to have a less direct impact on the infection transmission, leaving *R*_0_ effectively unchanged. Indeed, re-introduction of viruses in these models always led to population extinction when case fatality was high (e.g. 50%) (see the electronic supplementary material). Lastly, the introduction of a substantial population structure with small sub-population sizes and low demographic rates representative of non-human primates did not allow for the long-term persistence (e.g. more than 1 year for *SIR* and 3 years for *SEIR* models, see the electronic supplementary material), even in large populations (greater than 300 000), with a range of spatial coupling from very low (0.1) to strong (0.9) (see electronic supplementary material, figures 15 and 16), and with no disease-induced mortality. Here, the number of infected and therefore seropositive animals was small, with the infection spreading to few sub-populations. Up to ≈20% of a specific individual sub-population with ≈100 individuals within it might become infected at the epidemic peak, and up to ≈50% seroprevalence was reached within this sub-population; however, few sub-populations were infected and overall the population prevalence was very low (less than 0.05%), with the total seroprevalence reflecting this at less than 0.1%.

## 4. Discussion

The role that apes and other non-human primates play in *Ebolavirus* maintenance in Nature is uncertain. Previous work has modelled the impact of EVD on ape populations and their recovery [59]. Here, analyses of a series of transmission models suggest that EVD infection cannot be maintained in ape populations, even with repeated introductions [60]. Furthermore, the simple *SIR* model with high case fatality and repeated incursions produces similar outcomes to what appears to be seen in Nature, i.e. small to medium-sized clusters of cases with high case fatality and very low seropositive animals in the population. The low seropositivity was despite the analyses assuming lifelong immunity. Relaxing lifelong immunity may increase the likelihood of viral maintenance, but experimental data in non-human primates (>400 days in animals surviving experimental infection [61]), field data from people (40 years after the 1976 outbreak in Yambuku, Democratic Republic of Congo [62]) and non-human primate surveillance data [17] suggest that completely waning immunity and undetectable antibodies are unlikely.

Prolonged incubation periods is another mechanism that may increase the likelihood of persistence in a population for directly transmitted diseases, such as that seen with rabies [11]. Experimental studies, as well as human outbreak data, suggest that this is relatively short for EVD-causing viruses in non-human primates [21]. Two notable exceptions to this are the single report of sexual transmission approximately 530 days after infection through semen in the West African outbreak [13] and recent evidence of recrudescence after 5 or more years [57]. Here, including an incubation period with an average of 9 days did not qualitatively change the findings, probably because of the relatively short incubation periods compared with the slow reproductive rates and lifespans; however, nor did viral repeated re-introduction (*δ*), which might also simulate recrudescence and late sexual transmission. Recent pharmacodynamic models of antibody reactivity among human survivors suggest that EBOV antibody reactivity declines over 0.5–2 years after recovery, but that restimulation occurs, perhaps through ongoing replication of EBOV after recovery, and that this would explain why recrudescence may occur occasionally among individuals [63].

We performed numerous other sensitivity analyses and all models and parameter combinations were run with frequency, density and a mixture of density and frequency dependence (i.e. *q* = 1, 0 and 0.5 in equation (2.3)). Our analyses supported frequency-dependent transmission in African apes, because no mixed or density-dependent models replicated field data, mostly through very high seroprevalence in contrast to field data [17,18].

Other simplifying assumptions were made, including equal natural birth and death rates (*μ*), likelihood of breeding, susceptibility and homogeneous population mixing. Strongly seasonal breeding can impact infection persistence in populations [64], including filovirus maintenance in bat populations [65,66]; however, Central African ape reproduction is not strongly seasonal, and ageing patterns in non-human primates are unlike those in people, with little reproductive senescence [66], so these simplifications appear reasonable. Apes and many other non-human primates have strong social groups. However, in most cases, including in non-human primates, increased modularity in social networks probably decreases social infectious disease spread across the meta-populations [67], though the impact of increased but moderate mortality through culling in structured populations has been shown to counterintuitively increase prevalence [68]. We also assumed exponential waiting time distributions, since changes to these for latent and infectious periods may not significantly affect extinction frequencies [69] and, without strong evidence to the contrary [70], seem suitable for relatively long-lived wildlife. The inclusion of a second homogeneously mixing population did not alter the findings with mortality. Furthermore, the inclusion of weak or strong spatially structured meta-populations with small sub-populations with *SIR* or *SEIR* dynamics still failed to ensure viral maintenance even in the absence of any disease-induced death, and high case-fatality rates will only increase the likelihood of infection failing to pass from one social group to another [41]. The largest sero-survey demonstrates very low seroprevalence against EVD [17]; however, two studies provide evidence that seroprevalence may be locally high, from a few per cent reaching up to ≈30% [18,20]. These field findings were partly replicated by the metapopulation model, where overall seroprevalence was very low, but small outbreaks in sub-populations caused very isolated local dynamics, even without high mortality. Increasing complexity in contact structures and incubation periods, along with other meta-population structures, however, might be interesting in future models.

The assumptions in this model are partly made for parsimony and are partly because of an absence of data, which also prevents model fitting. For example, here EVD is treated as if it is caused by a single viral species; however, specific techniques to detect more than one virus (e.g. [17]) still suggest limited non-human primate EVD persistence. Mortality from EVD reduces the likelihood of transmission. However, in people it has been shown that they may transmit infection after death. To allow longer infection periods and explore the impact of slower mortality, mortality in the immune (*R*) class was simulated with high mortality (e.g. 50%) and this had a profound effect on the model populations. Re-introductions with high case-fatality rates and transmission (*β*) consistently drove the populations to extinction. This result suggests that at least some mortality may occur later or perhaps more likely that ongoing infection in apes occurs after death in a similar way to people to drive ape population declines [1,22]. Chimpanzees have been reported interacting with their dead, in a similar way to humans, thus increasing potential exposure to ebolaviruses after death [71–73]. However, generally stable populations in habitats undisturbed by people suggest that these population dynamics with high EVD mortality are not typical of apes or other non-human primates.

Various modelling approaches been taken to address different questions for human EVD. For example, the effects of the size of ‘spillover’ events (number of introductions) on the likelihoods of observing outbreaks [33] and the proportion of detected spillover events have been modelled, suggesting that perhaps half of all EVD events have not been reported [74]. However, most estimate *R*_0_ and model control interventions (e.g. [33,44,75]) for outbreaks with *R*_0_ >1, with varying degrees of complexity (e.g. spatial heterogeneity) introduced during and after the large West African outbreak owing to increasing numbers of cases [33,76–78]. How similar or different the dynamics are between people, apes and other non-human primates is not clear, but human density due to sociability might be enough to allow *R*_0_ > 1 at times.

New data will likely change our understanding of EVD ecology and the absence of definitive evidence for bats being the main ebolavirus reservoirs makes conclusive statements difficult [26]. However, here the model results suggest that wild non-human primates at relatively low population sizes and densities, in contrast to people, are unlikely to allow EVD-causing viruses to persist for prolonged periods because of the high case-fatality rates they suffer.

## Data accessibility

This article has no additional data.
